# A TALE OF TWO EXPERIENCES: NAVIGATING END-OF-LIFE CARE WITH A HISTORY OF INCARCERATION

**DOI:** 10.1093/geroni/igad104.1477

**Published:** 2023-12-21

**Authors:** Gabriel Lutz, Yulin Yang, Chixiang Chen, Raya Kheirbek

**Affiliations:** University of Maryland School of Medicine, Baltimore, Maryland, United States; University of California, San Francisco, San Francsico, California, United States; University of Maryland, Baltimore, Maryland, United States; University of Maryland School of Medicine, Baltimore, Maryland, United States

## Abstract

**Objectives:**

The negative health effects of being imprisoned are well-documented throughout one’s lifetime, but the impact of incarceration on end-of-life experience is not fully understood. Thus, this study aims to explore the relationship between a person’s history of incarceration and their end-of-life experience

**Methods:**

The Health and Retirement Study, a survey of Americans aged 50 and above that is nationally representative, provided the data for this study, which involved a secondary analysis of existing information. The dataset included individuals who had participated in the HRS but had deceased between 2012 and 2018.

**Results:**

Among 1710 respondents aged 78 (SD10 years) on average at baseline, there are 53% female, and 8% with incarceration history (n=139). We found incarceration history to be positively associated with caregiving and lower risk of dying alone OR 1.84 (95% CI: 0.64-5.21) higher chance of using hospice OR 1.16 ( 95% CI: 0.72- 1.87) and dying at home, but lower levels of quality of life at the end of life OR -0.44 (95% CI: -1.88, 1.01) and utilization of palliative care services OR 0.49 ( 95% CI: 0.21, 1.12).

**Conclusion:**

Our study, which included representative sample of older adults from across the country has revealed an association between a person’s past imprisonment and their end-of-life experience . The study highlights the pervasive effects of incarceration and underscores the importance of developing effective palliative care strategies for early identification of the needs to alleviate suffering and improve quality of life for this vulnerable population.

